# Acid Neutralization Capacity of a Tricalcium Silicate-Containing Calcium Phosphate Cement as an Endodontic Material

**DOI:** 10.6028/jres.115.033

**Published:** 2010-12-01

**Authors:** A. Maria Cherng, Shozo Takagi, Laurence C. Chow

**Affiliations:** American Dental Association Foundation, Paffenbarger Research Center, MML, National Institute of Standards and Technology, Gaithersburg, MD 20899

**Keywords:** acid neutralization, basicity, calcium phosphate cement, endo-materials, mineral trioxide aggregate, setting time, tricalcium silicate

## Abstract

A calcium phosphate cement (CPC) was shown to have the necessary attributes for endodontic materials except adequate basicity needed for antimicrobial properties. To enhance its basicity, tricalcium silicate (Ca_3_SiO_5_), a highly alkaline compound, was added to CPC at a mass fraction of 0.25, 0.5 or 0.75. The basicity, acid neutralization and physical properties of the CPC-Ca_3_SiO_5_ composites were investigated. Mineral trioxide aggregate (MTA) was used as the control. The acid neutralizing capacity of the CPC-Ca_3_SiO_5_ composites and MTA were measured by titrating the suspensions of ground set samples with a 0.2 mol / L HCl at predetermined pH levels, i.e., 11, 9.0, and 7.4. The setting time of CPC-Ca_3_SiO_5_ composites determined by the Gilmore needle method was 40 ± 10 min. Acid neutralizing capacity of CPC depended (p < 0.05) on Ca_3_SiO_5_ content. CPC containing 75 % Ca_3_SiO_5_ could neutralize slightly less acid than MTA (p < 0.05), but it had a shorter setting time than that of MTA (> 4 h) and excellent handling properties.

## 1. Introduction

Calcium hydroxide (Ca(OH)_2_) has been historically used as a major ingredient for pulp capping or to establish apical closure and avoid surgery in the apexification procedure [1]. The treatment requires high patient compliance, multiple appointments extending over a long period of time, and susceptibility to coronal leakage or fracture of the already thin root [2, 3]. Mineral trioxide aggregates (MTA) is currently thought to be biocompatible [4] and effective in the above procedures. MTA, however, has relatively poor handling properties, namely very long hardening times [5], little washout resistance (no coherence) and noinjectablility. Calcium phosphate cement (CPC) [6], a mixture of equimolar amounts of tetracalcium phosphate (TTCP) and dicalcium phosphate anhydrous (DCPA), has the above attributes needed for use in endodontic treatment, as well as good adaptation to the dentin walls and the ability to set without shrinkage. The mechanical strength of CPC is increased in saliva or plasma-like fluid [7]. *In vitro* [8, 9, 10], animal and clinical studies [11, 12] have been conducted to investigate the feasibility of using CPC for these treatments. Unmodified CPC materials do not typically have the same high alkalinity, needed for anti-microbial activity, as Ca(OH)_2_ or MTA. Tricalcium silicate (Ca_3_SiO_5_) is the major component of Portland cement, which has nearly the same composition as that of MTA. Tricalcium silicate is a highly alkaline compound due to its ability to release Ca(OH)_2_ [13]. In this study, various amounts of tricalcium silicate were added to CPC to enhance its basicity. Ca_3_SiO_5_ was selected instead of Ca(OH)_2_ because it has lower solubility. The basicity of an endodontic material can be expressed in terms of two different aspects: (1) the highest pH value obtainable and (2) acid neutralizing capacity. The highest obtainable pH value should represent the pH of the fluids at the surface or within the pores of the material under *in vivo* condition, and this value can be determined by measuring the pH of a physiological-like solution in contact with the material.

The acid neutralizing capacity represents the ability of the material to absorb acids produced by bacteria/inflammatory cells while holding the pH above a specific value. This quantity is expected to increase with decreasing pH. For example, the material should be able to neutralize a greater amount of acid if the pH is allowed to drop to pH 9 compared to pH 10. Thus, this quantity should be expressed as a function of pH rather than as a single value. At the present time, none of the acid neutralizing capacity has been investigated before. Hence, the objectives of this study were to evaluate the basicity, acid neutralization capacity and physical properties of CPC-Ca_3_SiO_5_ and MTA.

## 2. Materials and Methods

### 2.1 Materials and Instruments

CPC was made of equimolar TTCP (a mass fraction of 0.73) and DCPA (a mass fraction of 0.27). Ca_3_SiO_5_ was prepared using a sol gel method [14] by firing CaCl_2_ and tetra-ethyl ortho silicate (Sigma, St. Louis, MO).^1^ One hundred grams of Ca_3_SiO_5_ were ground in a 250 mL agate jar with five agate balls (30 mm diameter) for six min in a ball mill (Retsch PM4, Brinkman, NY) at 200 rpm. MTA was purchased from Dentsply (Johnson City, TN). The titrant was a solution containing 0.2 mol/L HCl and 150 mmol/L NaCl, the latter being used as a background electrolyte. Impulsomat 614 and Dosimat 665 Titrator (Brinkman Instruments, Westbury, NY) were used in the acid titration study, and a Universal Testing Machine (United Calibration Corp., Garden Grove, CA) was used to measure diametral tensile strength (DTS). Poly-R dye (Sigma, St. Louis, MO) was used as the indicator for testing the sealing ability of CPC cement and MTA. The phase present in the set samples was identified by powder X-ray diffraction (XRD) (DMAX 2200, Rigaku Denki Co., Ltd, Woodlands, TX).

### 2.2 Sample Preparation and Acid Titration/Neutralization

The powders were prepared by combining CPC with a mass fraction of 0 (CPC), 0.25 (CPC-1), 0.5 (CPC-2) or 0.75 (CPC-3) of Ca_3_SiO_5_. The cement liquid was 0.5 mol/L Na_3_PO_4_. CPC-Ca_3_SiO_5_ samples were prepared by mixing the powder and liquid at P/L = 2.65 to produce pastes with good workability. MTA samples were prepared by following manufacturer’s instructions (P/L = 3.3) and were used as the control. After mixing, each specimen was prepared by packing the paste into a stainless steel mold (6 mm d × 3 mm h), which was subsequently covered on both the top and bottom, with two fritted glass plates (4-mm thick, 38 % porosity) and incubated at 37°C and 100 % humidity to keep the sample moist but not directly in contact with water. After 24 h, the set sample was demolded, dried at 70°C, and stored in a capped glass vial. For the basicity and acid neutralization capacity measurements, the set sample was ground by a mortar and pestle, and 0.2 g of the sample was placed in 10 mL of 150 mmol/L NaCl solution under a N_2_ atmosphere (to prevent atmospheric CO_2_ incorporation) at 37°C. A flat end combination pH electrode (Thomas Scientific, model # S450CBNC, Swedesboro, NJ, USA) was connected to a pH meter/titrator, and the pH was measured continuously to determine the highest pH obtainable. The sample suspension was under constant stirring (350 rpm) and the pH of the solution was monitored. The standard uncertainty of the pH measurement was estimated to be 0.01 pH unit.

After the maximum pH was recorded, 0.2 mol/L HCl in 150 mmol/L NaCl solution was added to reduce the pH to predetermined levels (9.0 and 7.4), and the amount of acid titrated (consumed) in order to reach each of the levels was recorded. The data would be presented as mEq of acid neutralized per unit mass of the test sample as a function of pH. The standard uncertainty of the acid neutralization capacity measurement was estimated to be 0.02 mEq acid/g of sample.

### 2.3 Setting Time and Mechanical Strength

The setting time of each type of material was determined using the Gilmore needle method [15]. Diametral tensile strength (DTS) was used as a measurement of mechanical strength of the materials. For DTS measurements, each set sample was prepared as described above, placed in 1 mL of distilled water and incubated at 37°C and 100 % relative humidity for 24 h. The DTS of the wet set samples (6 mm d × 3 mm h) were measured using a Universal Testing Machine, as previously described [16]. The standard uncertainty of the acid neutralization capacity measurement was estimated to be the standard deviation of the measurement.

### 2.4 Phase Composition Characterizations

Set samples of CPC, CPC-1, CPC-2, CPC-3, and MTA both before and after titration experiments were also characterized by powder x-ray diffraction (XRD) to determine the phase composition.

### 2.5 Sealing Ability Tests

Sealing abilities of wet set samples of CPC, CPC-1, CPC-2 and CPC-3, Ca_3_SiO_5_ and MTA before titration were estimated by 1 % Poly-R dye penetration test [17]. The standard uncertainty of the dye penetration measurements was estimated to be 0.1 mm based on the uncertainty of the measuring ruler.

### 2.6 Statistical Analysis

ANOVA and Newman-Keuls multiple comparison tests were performed to analyze the setting time, DTS, and acid neutralization capacity data.

## 3. Results

The setting times (mean ± s.d.; n = 3) for CPC, CPC-1, and CPC-2 were 30 ± 3 min which were not significantly different (p > 0.05) but significantly shorter (p < 0.05) than that of CPC-3 (50 ± 5 min). However, CPC-3 had a significantly shorter setting time than that of MTA (240 ± 30 min). The DTS (mean ± s.d.; n = 6) for CPC, CPC-1, CPC-2, CPC-3 were 5.38 ± 0.75, 3.74 ± 0.57, 4.79 ± 0.34, and 5.17 ± 0.56 respectively, which were not significantly different (p > 0.05) yet lower than that of MTA (13.37 ± 0.23).

**Fig. 1:**
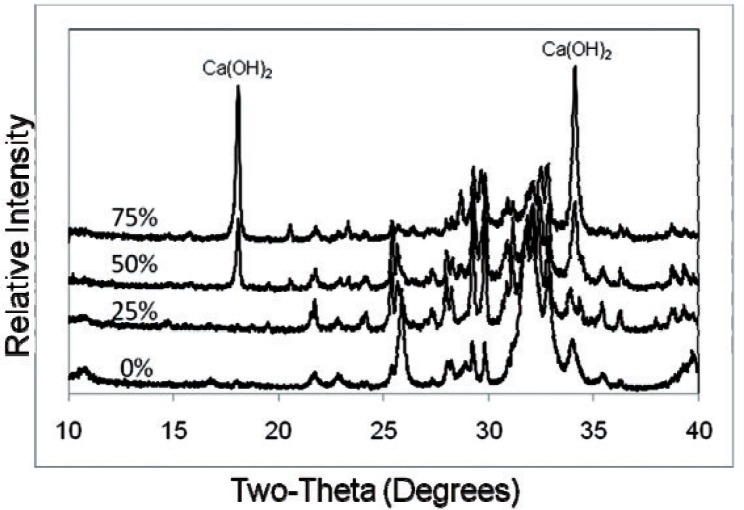
XRD patterns of CPC, CPC-1, CPC-2 and CPC-3 before titration.

**Fig. 2:**
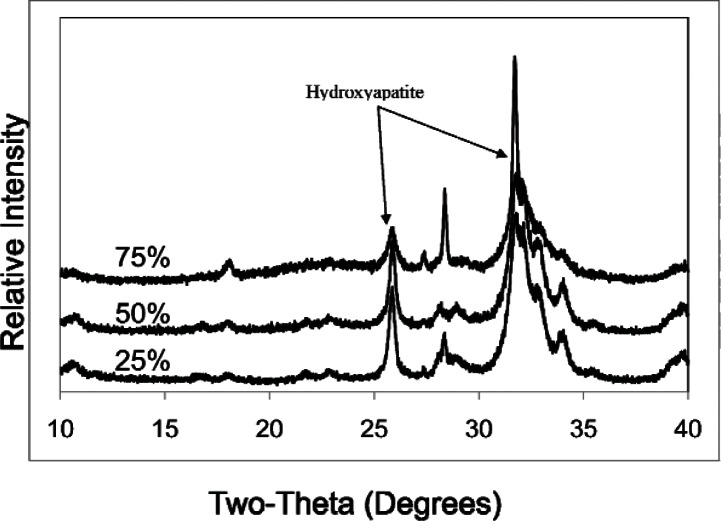
XRD patterns of CPC-1, CPC-2, CPC-3 after titration with 0.2 mol / L HCl.

**Fig. 3:**
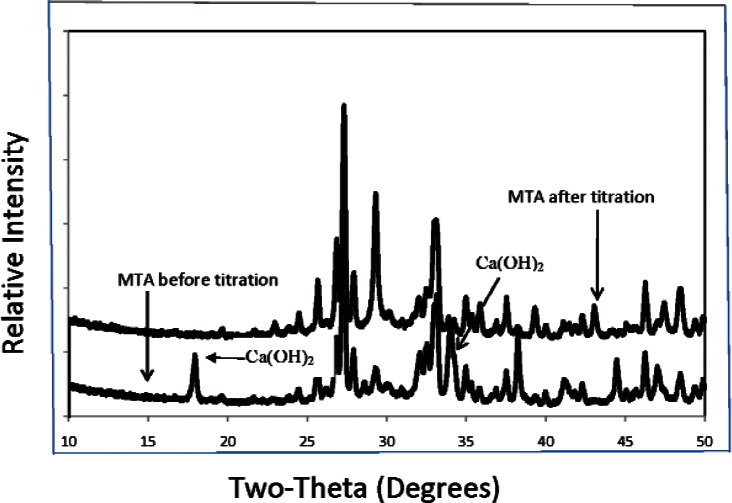
XRD patterns of MTA before and after titration with 0.2 mol / L HCl.

The highest obtainable pH and the amounts of HCl consumed (in mEq/g of sample) for CPC, CPC-1, CPC-2, CPC-3 and MTA (mean ± s.d.; n = 3) are shown in Table 1. The dye penetration test of CPC, CPC-1, CPC-2, CPC-3 (P/L = 2.65), Ca_3_SiO_5_ and MTA (mean ± s.d.; n = 2) indicated that the depth of penetration by measurement was completely penetrated, 0.2 ± 0.1, 0.1 ± 0.1, < 0.1, < 0.1 and < 0.1 mm respectively. Powder x-ray diffraction patterns of the CPC mixtures and MTA both before (Fig. 1 and Fig. 3) and after (Fig. 2 and Fig. 3) the titration revealed the presence of Ca(OH)_2_, which was largely consumed after acid neutralization.

## 4. Discussion

In this study, Ca_3_SiO_5_ was used as an alkaline additive instead of Ca(OH)_2_ because it dissolves more slowly and can improve the sealing ability of CPC as a filler. MTA contains bases (Ca_2_SiO_4_, Ca_3_Al_2_O_6_) other than Ca_3_SiO_5_ that may contribute to the higher acid neutralizing capacity at the levels of pH 9 and 7.4 (p > 0.05). Acid neutralizing capacity of the CPC-Ca_3_SiO_5_ composites depended strongly (p < 0.05) on the Ca_3_SiO_5_ content. CPC-3 (75 % Ca_3_SiO_5_ content) also had a relatively high neutralization capacity (p > 0.05), although MTA could neutralize more acid.

Basicity has been related to the antimicrobial property of endodontic materials. The capacity of acid neutralization by the material at different pHs should be an important property, but it has not been adequately addressed at the present time. There is neither a pre-set highest obtainable pH value nor a minimum acid neutralization capacity at a particular pH that has been established as the requirement for the antimicrobial activity. However, by combining different amounts of Ca_3_SiO_5_ with CPC, we can design CPC at different alkaline pHs if it is required.

The CPC-Ca_3_SiO_5_ samples had lower DTS (4.79 MPa) than MTA (13.3 MPa), likely due to the lower P/L ratio. However, in the area of endodontic application, strength is not as critical as that of restorative materials.

Powder XRD patterns of the CPC mixtures indicated the formation of hydroxyapatite which was found to adapt to the contours of the dentin surfaces at a microscopic levels. XRD also showed the level of Ca(OH)_2_ in CPC-Ca_3_SiO_5_ mixtures corresponding with the mass fraction of Ca_3_SiO_5_.

MTA samples prepared by following manufacturer’s instruction had a much longer setting time (4 h) than that of CPC-Ca_3_SiO_5_ samples (30 min to 50 min).

Although the P/L ratio used for the preparation of CPC-Ca_3_SiO_5_ samples was lower than that of MTA, it still provided an equally good seal against dye penetration and had good coherency and better handling properties than MTA.

## Figures and Tables

**Table 1:** Acid neutralization capacity of CPC, CPC-1, CPC-2, CPC-3 and MTA

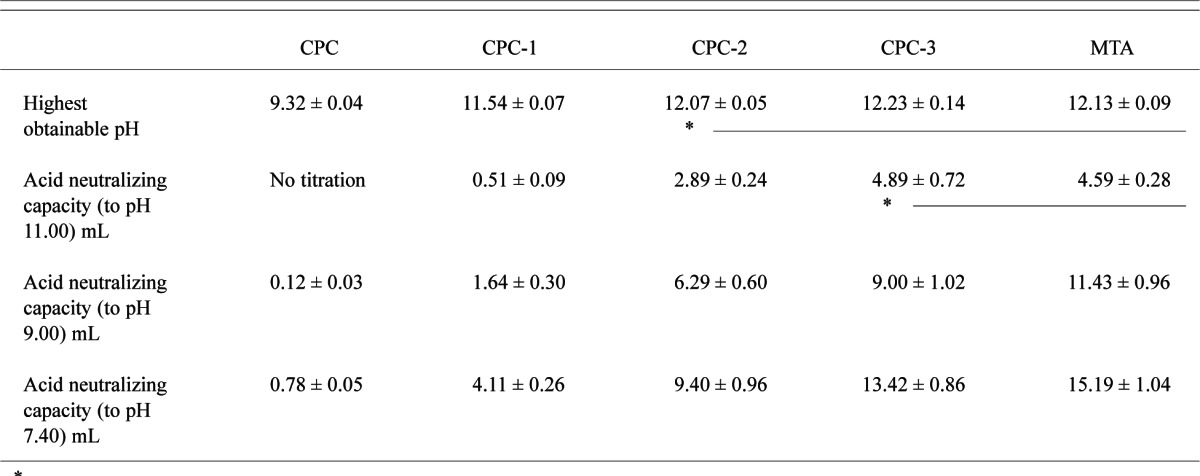

* Values connected by a line were not significantly different (p > 0.05).

## Footnotes

1 Certain commercial equipment, instruments, or materials are identified in this paper to specify the experimental procedure adequately. Such identification is not intended to imply recommendation or endorsement by the NIST or the ADAF, nor is it intended to imply that the materials or equipment identified are necessarily the best available for the purpose.
